# Development and evaluation of a novel 3D in-shoe plantar strain measurement system: STAMPS3D

**DOI:** 10.1177/09544119251330738

**Published:** 2025-04-12

**Authors:** Francesca Sairally, Rory P Turnbull, Heidi J Siddle, David A Russell, Claire Brockett, Peter R Culmer

**Affiliations:** 1 School of Mechanical Engineering, University of Leeds, Leeds, West Yorkshire, UK; 2 Leeds Institute of Rheumatic and Musculoskeletal Medicine, University of Leeds, Leeds, West Yorkshire, UK; 3 Leeds Vascular Institute, Leeds Teaching Hospitals NHS Trust, Leeds, West Yorkshire, UK; 4 Leeds Institute of Clinical Trials Research, University of Leeds, Leeds, West Yorkshire, UK; 5 Department of Mechanical Engineering, University of Sheffield, Sheffield, UK

**Keywords:** Diabetes, diabetic foot, strain, shear, pressure, plantar, DIC, Digital Image Correlation, stereo

## Abstract

The formation of diabetic foot ulcers (DFU) is consequential of peripheral neuropathy, peripheral arterial disease and foot deformity, leading to altered foot biomechanics and plantar loads. Plantar load comprises of normal pressure and shear stress, however, there are currently no in-shoe devices capable of measuring both components. The STrain Analysis and Mapping of the Plantar Surface (STAMPS) system, developed at the University of Leeds, utilises Digital Image Correlation (DIC) to measure the strain captured by a plastically deformable insole, as a method to understand plantar load during gait. A 2D DIC software was used to capture cumulative plantar strain and displacement pointwise data, however this method was limited to the analysis of planar surfaces. To address this, 3D instrumentation and DIC methods have been developed and implemented into the STAMPS3D system, used as a tool to capture data that is representative of the non-planar nature of plantar surfaces of the foot. A case-study is used to demonstrate how STAMPS3D can measure multi-dimensional strain, bringing potential to improve clinical screening of DFU risk.

## Graphical abstract

**Graphical abstract:**
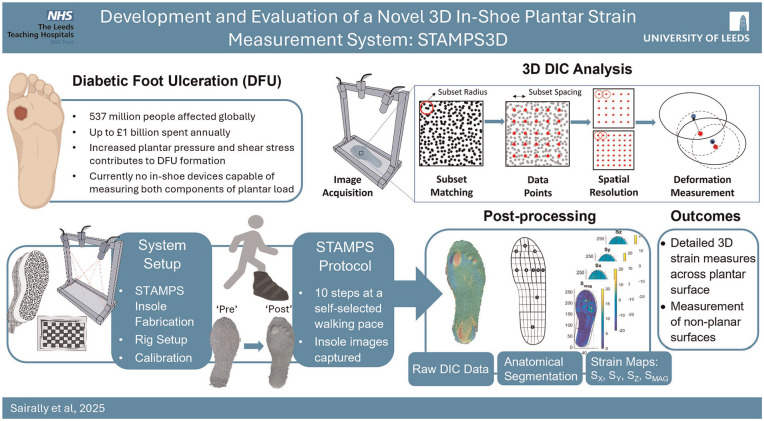

## Introduction

Diabetes is a globally prevalent chronic condition, with numbers expected to reach 537 million and 783 million by 2030 and 2045 respectively.^1^ Of those with diabetes, up to 25% will go on to develop a diabetic foot ulcer (DFU), with a 40% recurrence rate at 1 year in those that heal.^2,3^ Consequently, diabetic foot care has been estimated to cost nearly £1 billion annually based on NHS England spending from 2014/2015.^4^ To address the social and financial costs associated, the James Lind Alliance^5^ identified the top 10 diabetic foot research priorities. From this set of priorities, six of the ten included ‘prevention’ highlighting its importance in diabetic foot care. DFU formation is driven by diabetic peripheral neuropathy (DPN) and soft tissue changes, resulting in structural foot deformities and increased plantar load within these regions.^6^ Unfortunately, in the presence of neuropathy, there is a loss of the protective pain sensation which contributes to soft tissue damage due to repetitive high or abnormal stresses going unnoticed.

Plantar load comprises of pressure and tangential shear stress. Plantar pressure is the force acting perpendicular to the surface, whereas shear stress acts parallel to the surface with anterior/posterior and medial/lateral components.^7^ Previously, increased plantar pressure has been identified as a risk factor for DFU development. In regions where peak plantar pressure exceed 650 kPa, risk of ulceration has been described to increase by 6-fold, in contrast to areas below that pressure threshold.^8^ Additionally, plantar pressure has been found to be greater in those with diabetes and more so in those with DFUs present.^9^ However, the correlation between peak pressure and DFU formation is not definitive, as shown by Veves et al.,^10^ where although it was concluded that high plantar pressures were good indicators of ulceration (especially in the presence of neuropathy) only 38% of ulcers were identified at peak pressure locations. It has also been found that ulceration occurs at pressures that would be otherwise considered normal in people without diabetes, suggesting that other factors may contribute to DFU formation.^11^ Yavuz et al.^12^ found that there are two cycles of shear in contrast to pressure during a single step, due to stresses that arise in opposing directions during contact and push-off phases of gait. This suggests that shear may have a greater effect on plantar tissues, resulting in an increased risk of tissue damage and DFU formation under repetitive or elevated shear forces in contrast to the effects described under peak pressures. Although it has been suggested that pressure and shear may be influential in DFU formation, plantar shear is still poorly understood due to measures being difficult to capture. In addition to this, measurement technology is often limited to research conditions due to associated cost, time and expertise required for use.^13^

Currently, there are no commercial measurement systems capable of capturing both plantar pressure and shear. A custom measurement platform consisting of 80 sensors has been developed in a research setting and is capable of measuring both components of plantar load.^14,15^ However, this method is limited to barefoot conditions and therefore is not representative of in-shoe stresses that may arise during gait, as well as device size impeding clinical usage. In-shoe measurement devices for shear have also been described with sensors either being directly attached to the plantar surface of the foot or imbedded in an insole worn by an individual.^16–18^ In these cases, the spatial resolution is restricted by the number of sensors and therefore unable to capture data across the entirety of the plantar surface. In addition to this, sensors attached directly to the foot may disrupt natural gait patterns compared to those integrated within a wearable insole. Hence, the development of a wearable device capable of capturing in-shoe plantar load is necessary, ensuring that the limitations described are addressed.

At the University of Leeds, the STrain Analysis and Mapping of the Plantar Surface (STAMPS) system has been developed.^19^ This approach involves a plastically deformable insole, which utilises 2D Digital Image Correlation (DIC) to capture cumulative plantar strain across a period of gait. Consequently, real-time strain data is not captured, however this compromise enables higher spatial resolution of the data and mapping across the plantar surface. DIC is an optical technique which uses an applied speckle pattern to track local regions between deformed and undeformed images, which are used to calculate displacement and strain. Further technical details on the development and validation of STAMPS are presented in prior work.^19^ The STAMPS system has been successful in capturing plantar strain data in a healthy cohort, identifying a range of normal values across 18 healthy participants.^20^ Although the system was successful in capturing plantar strain data, the 2D nature limits its application to planar surfaces, which are not representative of the surface of the foot where DFU formation occurs. Hence, this paper investigates the implementation of 3D DIC methods to better represent the non-planar nature of the plantar surfaces of a foot, by capturing 3D strain measures. In doing so, improved accuracy of strain data indicative of plantar shear is expected to guide clinical screening procedures by providing quantitative measures to identify DFU risk, enabling appropriate management and aid in prevention.

This paper presents our work to extend STAMPS to a 3D measurement system (STAMPS3D) capable of measuring non-planar surfaces and capturing 3D strain data at the plantar interface. In System Development we present the development of the STAMPS3D approach, Experimental Evaluation of STAMPS3D details the experimental validation methods before a final Case Study with a single healthy participant demonstrating the differences between 2D and 3D methods. This is concluded with a discussion considering any associated limitations and future prospects for the described 3D methods.

## System development

### System requirements

The aim of this work is to progress the 2D DIC methods incorporated with the STAMPS system to instead harness open-source 3D DIC software and thus improve fidelity of measurement. The use of open-source software offers provides a cost-effective approach which enables future integration into semi-automated data analysis, and that can be readily adapted to stakeholder needs. Adopting this 3D DIC approach also requires development of stereo-camera instrumentation. The STAMPS3D system will then be capable of capturing data from both non-planar deformations and surfaces. The resulting 3D strain measures will then fully describe the *x*, *y* and *z* components of the strain tensors, providing an improved method in contrast to 2D DIC measures.

### 3D Digital Image Correlation (DIC)

STAMPS3D uses a combination of open-source toolboxes; 2D DIC is performed using ‘Ncorr’ (v1.2.2) which is then used by the ‘DuoDIC’ toolbox to form a 3D representation. Ncorr and DuoDIC are MATLAB (Mathworks, USA) based software. The main stages involved in 3D DIC, as implemented in DuoDIC, are: (1) Stereo Camera Calibration, (2) 2D DIC analysis (using Ncorr), (3) 3D reconstruction and (4) post-processing. These are illustrated in the Case Study provided in section ‘Case Study’.

This section now describes implementation of the 3D DIC technique, detailing configuration of the stereo imaging system, calibration of the system, selection of DIC parameters and finally the process of conducting and analysing the 3D DIC measures.

#### Stereo imaging rig design

**Figure 1.**
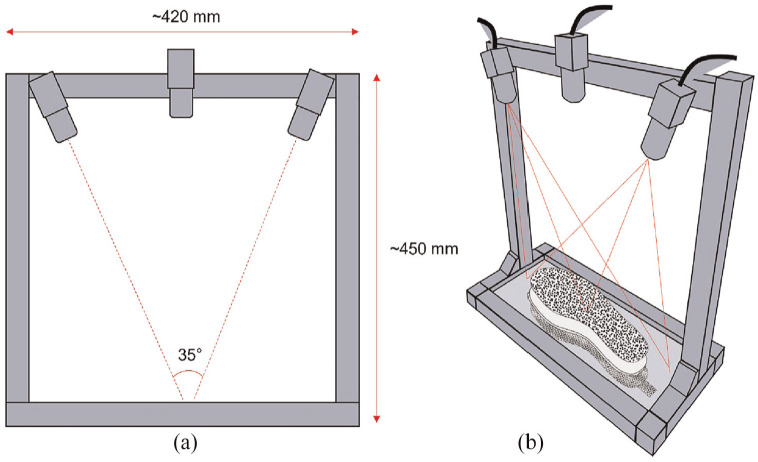
DIC camera rig setup, consisting of 2D and 3D configurations: (a) Left: Dimensions and relative position of cameras for each setup, and (b) Right: Combined field of view for the 3D setup.

A custom imaging rig was built to provide a consistent method of imaging insoles of various sizes before and after deformation. Both 2D and 3D camera setups were integrated to enable both DIC methods for comparative tests (Figure 1). Three high resolution USB cameras (Basler, Germany, A2A 1920-160UCBAS) paired with camera lenses (Basler, Germany, C23-08245M-P) with a fixed focal length of 8 mm were used to capture images with a resolution of 1920 × 1200. The system is powered and run through a high-performance laptop. For the 2D setup, a single camera is required and fixed central to the top of the rig frame, perpendicular to the base surface and at a height ensuring the entire base plate was within the field of view. For the 3D setup, two cameras acting as a single stereo camera pair were attached to the rig, aligned to the height of the single central camera (Figure 1, Left). Additional camera units can be used within this framework if occlusion of the 3D surface occurs, however two cameras were selected based on preliminary work which showed this configuration was appropriate in this context. Following established recommendations on good practice in DIC measurement for camera lenses used with a focal length of 8–12 mm,^21^ a stereo angle of 35° was chosen to ensure that entire base plate was within each camera field of view, such that the speckled insoles were fully visible and focussed across the whole insole (Figure 1, Right).

#### Calibration procedure

Calibration of the DIC system is an important aspect to determine camera parameters and the 3D configuration of the stereo camera pair. Using a rigid camera rig ensures that calibration is only necessary once, remaining unchanged unless the camera system is adjusted. A calibration procedure workflow has been developed to ensure each step is fulfilled to achieve accurate measures of the intrinsic and extrinsic parameters, which define the camera properties (focal length, optical centre and lens distortion) and relative positioning of the cameras in space (rotation matrix and translation vector) respectively.

Pre-calibration involved initialisation of cameras and lighting to prevent over-exposure or glare. Image acquisition was performed using a 15 × 15 mm checkboard calibration target, in which 66 image pairs were captured in a synchronised manner,^21^ such that images from each camera only differ in terms of their respective perspectives. The image pairs captured consist of rotation and translation of the calibration target about the *x*, *y* and *z* axes such that the entire field of view was imaged. Each of the images were quality checked prior to performing calibration to remove any images that were unfocussed or with glare. The MATLAB computer vision toolbox was used to calibrate the 3D system. Assessment of the calibration outputs (camera extrinsic parameters, reprojection error (RPE) and image rectification) was performed. Independent validation of the camera extrinsics was performed using a Qualisys motion capture kit. Calibration images were included such that they each have RPE < 1,^22^ with the average RPE ≤ 0.5 to ensure accuracy. Lastly, the image rectification provided within the MATLAB window was assessed to ensure the images corresponding to each camera were correctly row aligned. The successful calibration file was then used during 3D reconstruction, where any lens distortion was removed from each of the speckled images automatically. This is unlike DIC software, in which lens distortion must be removed manually from each of the images using a MATLAB script.

#### DIC parameter selection

**Figure 2.**
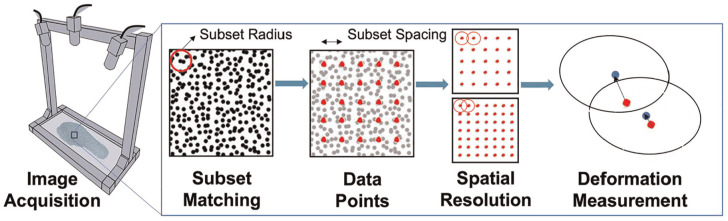
DIC analysis process including image acquisition, DIC parameters selection, spatial resolution and deformation measures demonstrated across two overlapping subsets.

The second step of STAMPS3D involves performing initial 2D DIC analysis. For this, the appropriate DIC parameters, including subset radius and subset spacing, was selected. The subset radius is a defined region consisting of a speckled pattern based on the diameter chosen. The speckles that fall within this radius are then used to match regions between the undeformed and deformed images across the insole. The subset spacing is the number of pixels defined between the datapoints, where measurements are captured. Each of these datapoints are located at the centre of a different subset, therefore overlap of subsets can be found where the subset spacing is less than the subset radius. The subset spacing chosen effects the spatial resolution of the data collected, where a smaller spacing results in a greater number of data points placed across the sample and therefore increasing the spatial resolution, demonstrated in Figure 2.

The recommendations on DIC parameter selection vary among the literature and are often subjective. Supplementary work to the 3D methods was performed to determine appropriate DIC parameters using an objective approach, ensuring that spatial resolution, subset distinguishability and computational processing time were considered. DuoDIC is a 3D extension of the 2D DIC software Ncorr, utilising it for step two where 2D DIC analysis is performed. Hence, the DIC parameters determined in the supplementary work were also used for this application. A subset radius of 17 pixels and subset spacing of eight pixels were selected. The 2D DIC analysis was performed for the images corresponding to each camera in the stereo pair, producing a set of 2D image points where the associated 2D measures are located.

#### 3D DIC analysis

The calculated set of 2D image points are used with the calibration file to reconstruct 3D image points. The final step involves the calculation of displacement and 3D sample deformations. The DuoDIC software also enables the removal of rigid body motion (RBM) from measures to account for misalignment of a given sample between imaging pre- and post-deformation, mitigating any mis-alignment of insoles between imaging phases.

#### Segmentation and post-processing

An analysis process was developed (Mathworks, USA) to process and provide visualisation of the computed deformation data. Firstly, the normal strains *S_X_*, *S_Y_* and *S_Z_* were extracted from the Green-Lagrangian strain tensor as calculated from the 3D DIC analysis. The corresponding strain map is segmented corresponding to ten anatomical regions of the foot according to a masking protocol employed by the commercial plantar analysis software (PEDAR INC., Novel GmbH, Munich, Germany): Heel, midfoot, 1st Metatarsal Head (MTH), 2nd MTH, 3rd MTH, 4th MTH, 5th MTH, hallux, second toe, toes 3–5. Based on the insole shape and size, the mask is scaled and adjusted to fit the individual plantar data. Strain data is allocated into each of the corresponding regions. Anatomically, the normal strains can be interpreted as the mediolateral axis (*S*_ML_), anteroposterior axis (*S*_AP_), pressure axis (*S*_P_) and strain magnitude (*S*_MAG_). The upper 10% *S*_MAG_ measures are highlighted to identify the global strain distribution about the localised peak strain measures.

### STAMPS3D insole fabrication

**Figure 3.**
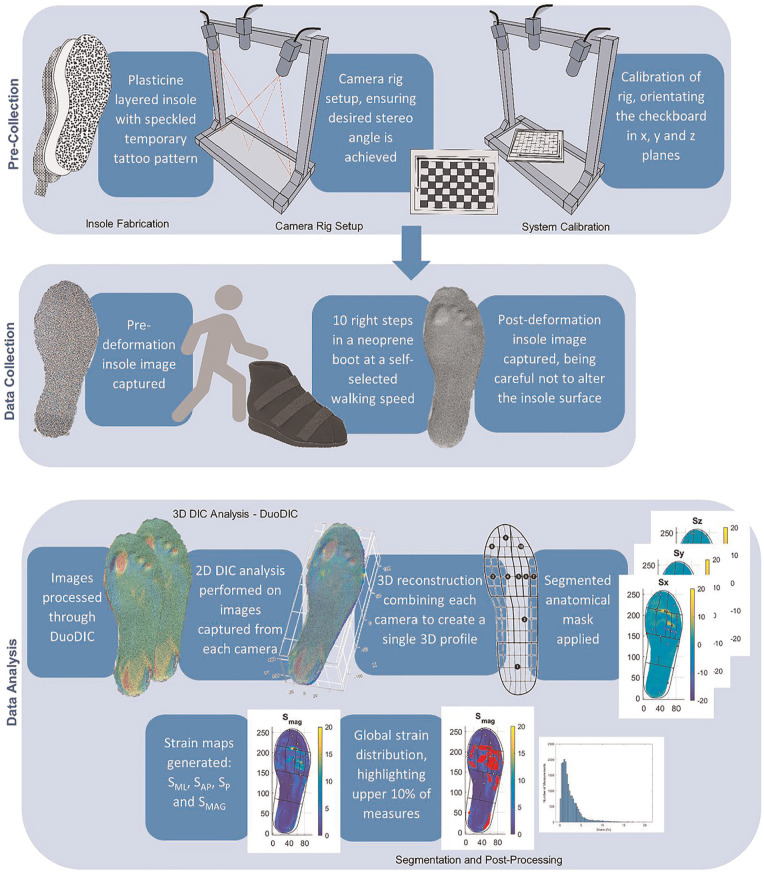
STAMPS3D procedure including, pre-collection preparation, data collection and analysis.

The STAMPS insole has a multi-layer structure, with a 5 mm mid-layer made of industrial plasticine to enable plastic deformation, which can be cut to measure a range of shoe sizes and shapes.^19^ A high-contrast speckle pattern is printed onto temporary tattoo paper using a commercial inkjet printer and adhered to the surface of the plasticine mid-layer using the water-activated sheet backing. The speckled pattern is generated using a commercial pattern generator (Correlated Solutions Inc.) defined with 65% speckle density, 0.8 mm speckle diameter and 75% pattern variation. Figure 3 outlines the steps involved in the insole fabrication, calibration, data capture and analysis for STAMPS3D.

### Experimental evaluation of STAMPS3D

Experimental testing was performed to validate the STAMPS3D system as a measurement technique. The tests aimed to first validate 3D deformation measurements on both planar and non-planar samples, comparing deformations and global surfaces of non-planar samples measured using 3D DIC (DuoDIC) against 3D scans (Artec 3D, USA), which were taken to be the benchmark. The second aim was to validate the shear measures captured using 3D DIC.

#### Methods

Testing was performed through indentation and shear tests using a universal load tester (Instron, 5943) and a custom multi-axis load tester (Thorlabs, NJ, USA), respectively. Speckled plastically deformable samples (50 × 50 mm) were made, consistent with the fabrication of the STAMPS3D insoles. For the planar case, a custom fixture was 3D printed (Form 3, FormLabs) to act as a supportive backing and prevent any unwanted warping during testing. For the non-planar case, samples were moulded into a 3D printed hemi-spherical surface with an inner diameter of 80 mm. Hemi-spherical indenters were 3D printed with diameters 20 and 30 mm. Indenter sizes were selected to test a range of deformations reflective of anatomical regions of the foot, such as the metatarsal heads, together with overlying soft tissue. An initial pre-load of 0.01 N was applied. Samples were then indented at a depth of 2 mm. Five repeats of each indenter size were performed (*n* = 15). Stereo images were captured for each test, pre- and post-deformation, and were used for 3D DIC analysis. 3D scans of samples were taken post-deformation. A custom script was developed to post-process the data and apply a sphere fit to either the local deformation or global surface of non-planar samples. From this an estimated radius of curvature was calculated with the corresponding root-mean-squared (RMS) error. The mean RMS error and standard deviation across the five samples were collected. These measures were compared against the 3D scan data, with percentage error for estimated radius of curvature and RMS error calculated.

For the shear tests, planar samples were fabricated as described above. Each sample was held in a custom 3D-printed fixture, which could be attached to the Thorlabs multi-axial stage, securing samples and ensuring shear occurred in a single axis. The 20 mm hemispherical indenter was used to indent the samples to a depth of 2 mm, followed by shear distances of 0, 1 and 2 mm. With five repeats for each test performed (*n* = 15).

**Figure 4.**
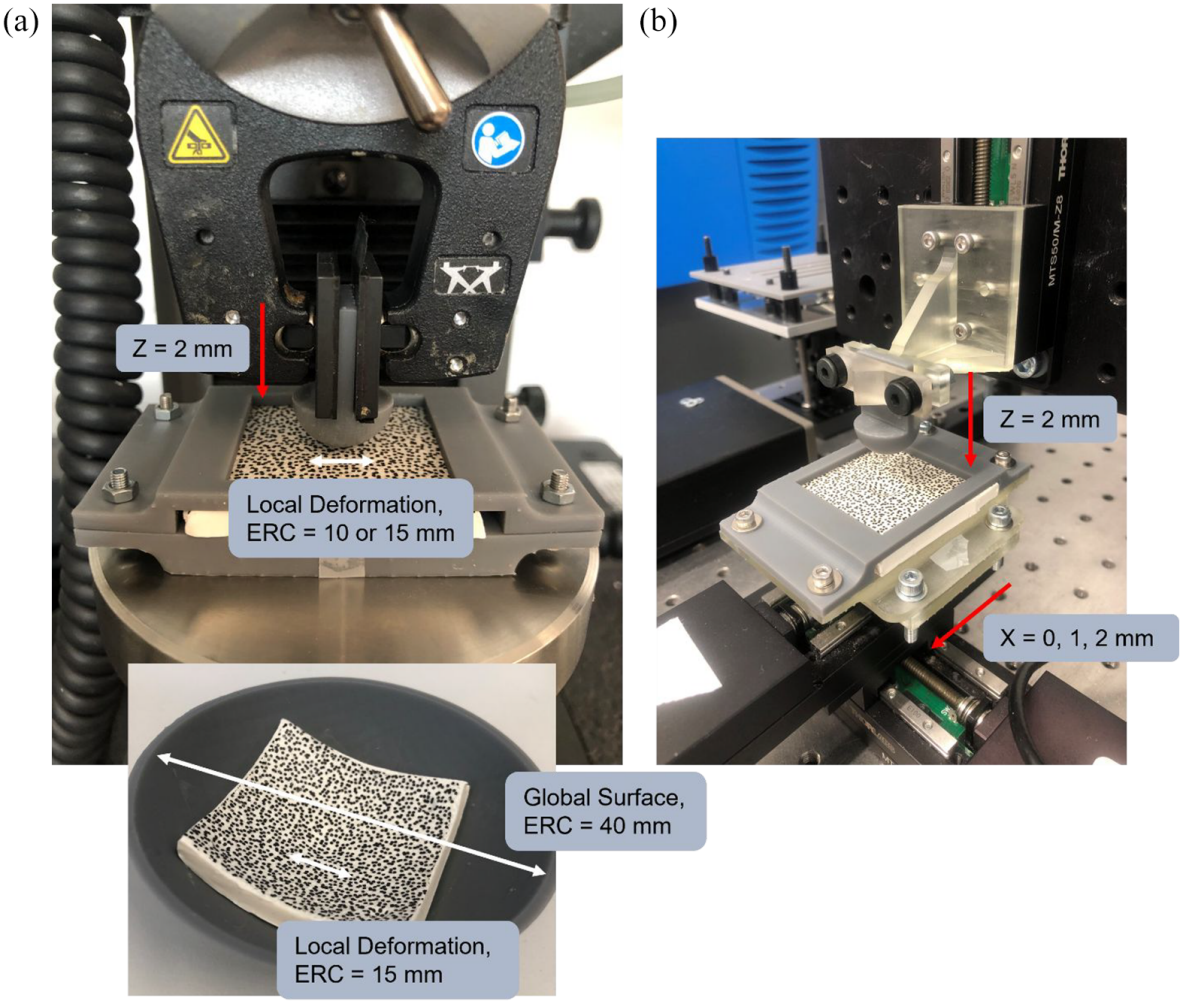
Method setup corresponding to: (a) indentation tests performed at 2 mm on planar (top) and non-planar samples (bottom) to validate simple geometry of deformations and global surfaces with expected radius of curvature (ERC) = 10, 15 and 40 mm and (b) shear tests performed at distances 0, 1 and 2 mm after an initial 2 mm indentation.

The test methods for each setup are demonstrated in Figure 4.

#### Results

**Figure 5.**
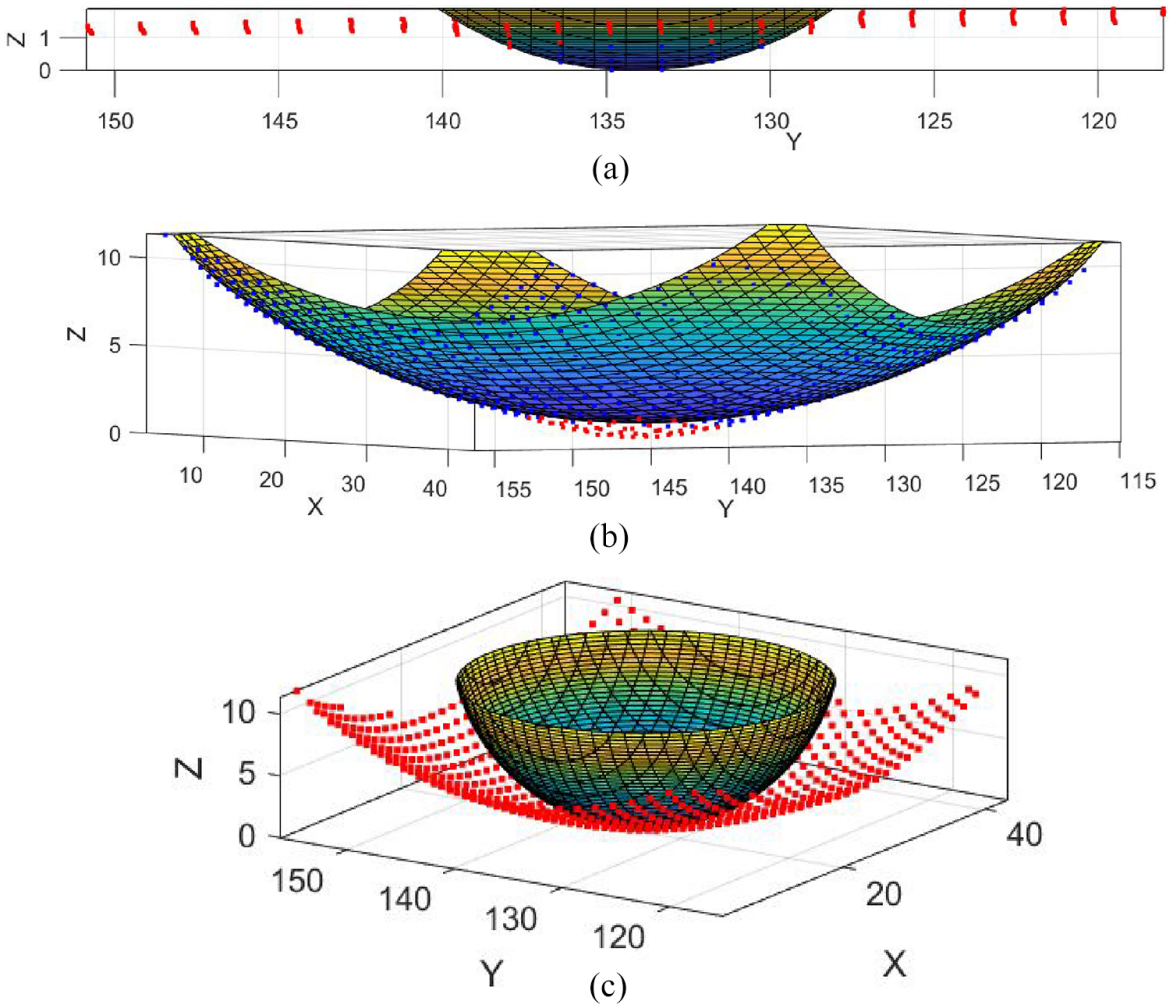
Examples of sphere fits applied to: (a) planar samples, (b) global surface of non-planar samples (excluding data points corresponding to local deformation), and (c) local deformation of non-planar samples (excluding data points which fall outside the deformation).

**Figure 6.**
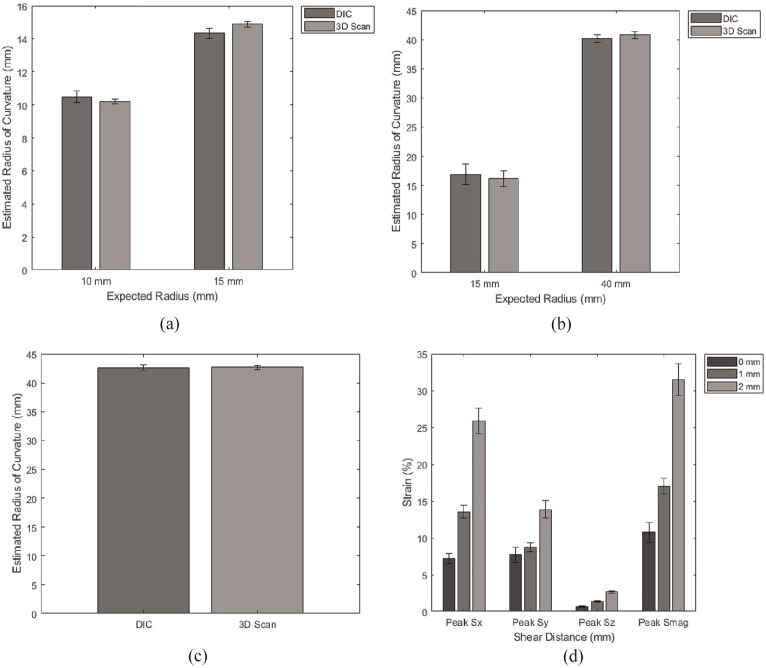
Average estimated radius of curvature for: (a) planar samples with local deformations using 10 and 15 mm radius indenters, (b) non-planar samples with local deformations using 15 mm radius indenter and global surface with 40 mm radius, (c) non-planar samples undeformed to measure global surface alone, and (d) peak strain measures for varying shear distances of 0, 1 and 2 mm (error bars ±95% confidence interval).

All tests described were successfully performed and analysed using 3D DIC. The first set of tests set out to validate simple geometry deformations. Sphere fits were applied as exemplified in Figure 5. Figure 6(a) to (c) compares the estimated radius of curvature corresponding to each indenter and global surface measured using either 3D DIC or a commercial 3D scanner. Figure 6(d) presents peak strain components *S_X_*, *S_Y_*, *S_Z_* and *S*_MAG_ reported for shear distances 0, 1 and 2 mm. Tables 1 and 2 report all the measures corresponding to those summarised in Figure 6, respectively.

**Table 1.** Average RMS ± standard deviation corresponding to sphere fit applied to DIC and 3D scan data for both planar and non-planar samples.

*Sample*	*Expected radius (mm)*	*DIC sphere fit RMS*	*3D scan sphere fit RMS*
*Planar*	10	0.015 ± 0.003	0.033 ± 0.003
*Planar*	15	0.008 ± 0.001	0.032 ± 0.005
*Global*	15	0.003 ± 0.001	0.018 ± 0.003
*Global*	40	0.081 ± 0.013	0.140 ± 0.019
*Global (undeformed)*	40	0.060 ± 0.007	0.096 ± 0.009

**Table 2.** Summary of average peak strain ± standard deviation using DIC, showing the effect of shear distance on the components of peak strain (%).

*Peak strain measures (%)*	*Shear distance (mm)*		
	0	1	2
*Peak S_X_*	7.169 ± 0.799	13.540 ± 0.985	25.850 ± 1.708
*Peak S_Y_*	7.718 ± 1.140	8.750 ± 0.675	13.875 ± 1.276
*Peak S_Z_*	0.706 ± 0.124	1.395 ± 0.097	2.665 ± 0.132
*Peak S_MAG_*	10.720 ± 1.526	17.027 ± 1.233	31.478 ± 2.131

The results show that the estimated radius of curvature corresponding to both local deformations and global geometry for the data captured using 3D DIC, closely align to the data captured using the 3D scanner (Figure 6). The average error across the three test configurations ranged between 0% and 5% where DIC measures were compared to the benchmark 3D scans. The average RMS for sphere fits applied to data analysed using 3D DIC were slightly lower than those of the 3D scans across each test configuration, in the range of 0.003–0.081 and 0.018–0.140 respectively (Table 1). Statistical significance between the DIC and 3D scan sphere fits were assessed using a paired *t*-test, where a significant result was determined if *p* < 0.05. Significant differences were determined for planar samples indented using 15 mm radius indenter and non-planar (deformed) samples for the 40 mm global surface (*p* = 0.02 and *p* = 0.002).

The results also show an increase in strain as shear distance was increased from 0 to 2 mm and was consistent across each of the peak measures *S_X_*, *S_Y_*, *S_Z_* and *S*_MAG_ (Figure 6(d) and Table 2). For shear distance = 0 mm peak *S_X_* and *S_Y_* were not found to be significantly different (*p* > 0.05), however statistical differences were found as shear distance was increased. The peak *S_X_* measure were found to be larger at shear distances 1 and 2 mm compared to peak *S_Y_* (*p* = 0.002 and *p* = 0.0002 respectively) and peak *S_Z_* (*p* = 0.00001 and *p* = 0.000009 respectively) which was expected due to the shear being applied in the *x*-axis. The peak *S*_MAG_ measures were larger than the other measures reported due to the cumulative effect of the *x*, *y* and *z* components of strain. These results were expected based on the experimental work reported in the previous STAMPS paper.^19^

## Case study

A case study was conducted to compare the use of 3D DIC methods in STAMPS3D with a 2D DIC approach, evaluated through use of the system with a healthy participant.

### Methods

Ethical approval was obtained from the University of Leeds Ethics Committee to conduct the case study (EPS FREC 2024-1462-1817). Eligibility included the participant being aged >18 years, capable of walking for 50 m unaided, and not diagnosed with diabetes or foot health conditions. Prior to assessment, the participant read the participant information sheet and provided written consent.

A STAMPS insole and supportive neoprene boot (Ninewells Boot, Chaneco Inc.) was selected based on the participant shoe size. A pre-deformation image of the STAMPS insole was taken using both the 2D and 3D camera setups of the rig. This was then inserted into the corresponding boot, with a similarly sized insole placed into the opposite foot to minimise any alterations in gait due to depth differences. The participant was asked to walk 20 steps, ensuring that ten steps were taken with each foot at a normal walking speed of their choice. The insole was removed and a ‘deformed’ image was taken again with both the 2D and 3D camera setups. Three repeats were taken. Both global and anatomical regions of the foot for measures of *S*_ML_, *S*_AP_, *S*_P_ and *S*_MAG_ were captured, in which *S*_P_ was only calculated using 3D methods. Two measures of *S*_MAG_ were also calculated using 3D methods, corresponding to those calculated using *x*, *y* and *z* components or *x* and *y* alone. *S*_MAG_ calculated using 2D methods corresponds to the latter. This post-processing analysis was performed using the custom processing described previously. Anatomical regions of the foot were segmented using a mask containing ten regions: Heel, midfoot, 1st MTH, 2nd MTH, 3rd MTH, 4th MTH, 5th MTH, hallux, second toe, toes 3–5.

### Results

**Figure 7.**
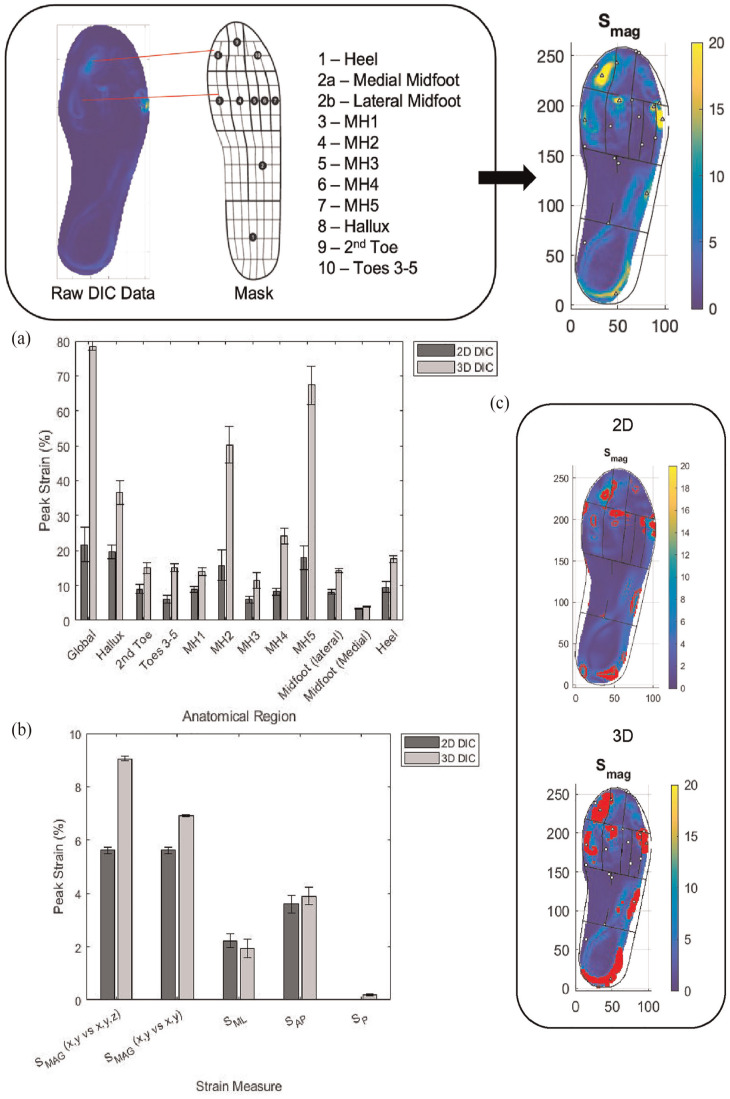
Demonstration of raw data masking and summary of results comparing 2D and 3D methods: (a) comparison of average peak *S*_MAG_ for deformed samples walked on for ten steps at a self-selected speed, (b) upper 10% percentile threshold of global strain measures showing differences in data distribution, and (c) examples of highlighted upper 10% S_MAG_ across anatomical regions of the foot (error bars ±95% confidence interval).

The regional peak *S*_MAG_ calculated using 2D and 3D DIC methods are reported in Table 3. Figure 7 demonstrates the masking of raw data and summarises the peak *S*_MAG_ data across anatomical regions for both 2D and 3D methods from Table 3, in addition to the percentile data highlighted in the corresponding strain maps.

**Table 3.** Average peak S_MAG_ ± standard deviation captured using 2D and 3D DIC methods across anatomical regions of the foot for deformed samples and corresponding.

*Region*	*2D*	*3D*	*3D*
	Peak *S*_MAG_ (*x,y*) (%)	Peak *S*_MAG_ (*x,y*) (%)	Peak *S*_MAG_ (*x,y,z*) (%)
*Global*	21.72 ± 1.08	61.44 ± 9.33	78.47 ± 18.82
*Hallux*	19.59 ± 0.81	32.56 ± 1.28	36.60 ± 1.41
*2nd toe*	8.96 ± 1.00	12.99 ± 1.68	15.03 ± 2.27
*Toes 3–5*	6.08 ± 1.94	12.78 ± 4.64	15.05 ± 5.35
*MH1*	8.85 ± 2.03	12.02 ± 6.13	14.03 ± 6.03
*MH2*	15.82 ± 4.79	44.91 ± 19.44	50.25 ± 27.20
*MH3*	5.97 ± 2.93	9.82 ± 5.22	11.33 ± 6.74
*MH4*	8.24 ± 1.38	17.78 ± 3.61	24.21 ± 3.56
*MH5*	18.07 ± 0.94	47.62 ± 2.35	67.30 ± 3.17
*Midfoot (lateral)*	8.14 ± 0.49	12.24 ± 1.45	14.25 ± 1.53
*Midfoot (medial)*	3.39 ± 4.49	3.52 ± 17.52	4.04 ± 19.57
*Heel*	9.52 ± 0.10	15.28 ± 0.57	17.59 ± 0.67

Figure 7(a) compares the average peak *S*_MAG_ (*x,y* vs *x,y,z*) computed using 2D and 3D DIC methods across the same samples after being worn for ten steps, reported in Table 3. The results show that across the anatomical plantar regions, similar patterns arise for both 2D and 3D methods. However, the strains produced using 3D DIC are larger than those calculated using 2D DIC. Statistically significant differences were found for all anatomical regions except at the 2nd toe, MH3, MH5 and medial midfoot (*p* < 0.05).

The upper 10% percentile of global strain measures were also recorded to assess strain distribution across anatomical regions of the plantar surface of the foot. The upper 10% threshold for 2D and 3D DIC methods for each strain measure is reported in Figure 7(b), with *S*_MAG_ measures highlighted in red across the strain maps in Figure 7(c). The results show that the upper 10% threshold for *S*_MP_ and *S*_AP_ were similar for 2D and 3D methods. Differences were found between the two methods for *S*_MAG_ (*x,y* vs *x,y,z*) and *S*_MAG_ (*x,y* vs *x,y*), with 3D methods resulting in larger threshold measures in both cases. The strain maps from Figure 7(c) show larger regions highlighted for the 3D methods due to increased magnitude shown in Figure 7(a). Increased detail of anatomical regions of the foot can also be seen in Figure 7(c), where 3D methods have been used, unlike the 2D strain map, which appears to have a smoother surface.

## Discussion

The development of a low-cost measurement system capable of measuring in-shoe plantar shear and plantar pressure has been reported, with STAMPS3D building on previous work to capture 3D plantar measurements. The total setup cost for the system is <£1000 with ongoing consumable costs of approximately £2.50 per insole. This makes STAMPS3D an affordable proposition which differs from other plantar measurement devices (e.g. Pedar, Novel) with potential to be used in low resource settings. The system weighs ca. 2 kg, with compact dimensions (see Figure 1), making it portable and easy to transport between clinical settings. The previous STAMPS study demonstrated that the strain measured using DIC resulted from normal pressure and tangential shear stress, where increased levels of the latter resulted in increased strain measures.^19^ Therefore, this presented a measurement technique capable of capturing in-shoe strain measures indicative of plantar pressure and plantar shear, where other techniques offer strictly pressure measurements, capture un-shod conditions or limited data resolution.^14–17,23^ However, the utilisation of 2D DIC methods means that measures are limited to a single plane and, therefore, may not accurately capture data from non-planar surfaces, such as plantar surfaces of the foot. This work reports validation of the adapted 3D measurement system (STAMPS3D), which utilises 3D DIC together with the previously reported STAMPS insole. The development of a low-cost 3D system aims to improve the accuracy of plantar load measurement at the foot-surface interface to guide risk identification and thus prevent DFU formation.

The results of the geometry tests showed that 3D DIC accurately measures deformations, with comparable accuracy to measurements from a commercial 3D scanner. Errors corresponding to the estimated radius of curvature reported between the two methods were 0%–5%. The average RMS, reported in Table 1, were slightly lower for the sphere fits applied to the DIC data compared to those of the 3D scans across each test configuration. This showed that for each test, the fitted spheres were closely matched to the selected data for either the local deformations or global surfaces. Differences in the radius of curvature estimation and sphere fit RMS errors may be due to the initial manual cropping of sample meshes from the 3D scanned data, which is necessary to select the specific data corresponding to the local deformations or global surface. Although two indenters of diameters 10 and 15 mm were used, indentation depths tested were limited to 2 mm. Preliminary tests showed that depths less than 2 mm resulted in inaccurate measures due to small deformations, including too few data points once processed using DIC or the 3D scanner. For depths greater than 2 mm, sample warping occurred. It is believed to occur due to full contact across samples not being present, as would be the case when participants wear STAMPS insoles.

Shear tests showed results that agreed with the UMT tests performed in the STAMPS study. The results showed that as shear distance was increased, the measured strain using 3D DIC increased. This was consistent for the magnitude of strain and each of the *x*, *y* and *z* strain components. For shear distance = 0 mm, peak *S_X_* and *S_Y_* were not found to be significantly different to each other with average strains 7.71% and 7.17%. These measures were expected due to the spread/shearing of material around the indenter in both the *x*- and *y*-directions, as deformation occurs at a single point in the *z*-direction. As shear distance was increased to 1 and 2 mm, an increase in all components of strain was found, however this was greatest for the *x* component. This was found since shearing of the samples were performed in the *x*-direction. The strains measured for *S_Y_* and *S_Z_* were lower than that seen of *S_X_*, particularly for *S_Z_* due to deformation occurring in that direction during initial indentation. However, the combined effect of each component can be seen for the peak magnitude of strain, *S*_MAG_. For these tests, normal force was not set as a control and so the results may represent the effect on strain as shear distance and normal force were increased together.

The STAMPS3D system was successfully used to measure plantar strain in the single-participant study, involving ten steps at a self-selected walking pace, following established protocol outlined in the STAMPS2D study.^19^ This study involves a small sample size to only demonstrate the application of STAMPS3D in contrast to 2D DIC methods rather than providing strain data representative of the population. Therefore, broader conclusions can not be made based on this data. Similar patterns could be seen from Figure 7(a) across each anatomical region for both 2D and 3D methods. However, 3D measures were larger overall due to the additional *z* component captured. Statistical differences were found for the regions, Global, Hallux, Toes 3–5, MH1, MH2, MH4, Midfoot (Lateral) and Heel. The remaining regions reporting no significance may be due to reduced contact between the foot and the insole (e.g. medial midfoot) or positional error during the data masking. The upper 10% percentile of strains across all anatomical regions were also reported to consider the data distribution across the foot, alternatively to using a single peak value as an indicator of high-strain regions. Figure 7(b) summarises the upper 10% percentile threshold of global strain measures, comparing the difference between 2D and 3D methods. No significant difference was found between 2D and 3D methods for *S*_ML_ and *S*_AP_, which suggests comparable distribution characteristics regardless of method used. Peak measures of *S*_ML_ and *S*_AP_ were significantly larger when 3D DIC was performed, highlighting that 3D measures are more complete since the distribution of data is comparable to 2D data, whilst also providing larger peak strain measures that are more representative of the non-planar surface of the foot. The upper 10% percentile threshold for both *S*_MAG_ measures were significantly increased where 3D methods were used. This is due to larger peak measures and the additional *S*_P_ component, as mentioned previously.

This system allows for adaptations based on user requirements unlike other insole measurement devices available which often have set sizes. The base of the rig is designed such that it can accommodate insole sizes up to a UK size 14 and insole shapes can be easily adapted to fit individuals with wider feet. In a healthy study consisting of 18 participants it was reported that no statistically significant relationship was found between *S*_MAG_, peak plantar pressure and the individuals weight.^20^ Therefore design changes to the system would not be needed and has the potential to be used across the wider population. In the current system, no modifications are required for participant use, however future work looks to use the STAMPS3D system on a diabetic cohort with varying degrees of DFU. Data collection can also be completed within 10–15 minutes making the system suitable for clinical application, fitting within patient appointment slots. Limitations of the STAMPS system have been previously reported.^19^ Although this work demonstrates the system’s ability to capture non-planar data, the STAMPS insoles themselves are planar. Future work will evaluate STAMPS3D using non-planar STAMPS insoles to enable full plantar contact. This will involve moulding the planar STAMPS insoles to the surface of contoured (non-planar) orthotic insoles to identify whether changes in insole characteristics such as stiffness, thickness or regions of variable stiffness prescribed to individuals, result in altered plantar strain measured. This could be useful to indicate the interaction between the plantar surface of the foot and contoured orthoses used to manage callus and high-risk areas for DFU formation.

## Conclusions

This study demonstrated that 3D DIC can accurately measure deformations on planar and non-planar surfaces, comparable to a commercial 3D scanner. The relationship between strain measured using DIC and shear have been shown, aligning with a previous study that validated a 2D system. This work provides evidence that STAMPS3D can capture relevant information and characterise plantar strain. The use of 3D DIC in the case study showed similar distribution patterns to that of the 2D system, and an overall increase in strain was measured due to being able to capture the *z* component of the strain tensor. Overall, using 3D DIC techniques here provides a higher fidelity measurement to assess in-shoe plantar loads, and provides for inclusion of non-planar insole surfaces typical of clinical situations related to diabetic foot ulceration
